# Hidden Blood Loss in Biportal Endoscopic Versus Traditional Open Spine Surgery: A Retrospective Comparative Study of Risk Factors and Clinical Relevance

**DOI:** 10.3390/jcm15051918

**Published:** 2026-03-03

**Authors:** Hyoung-Sik Kim, Sub-Ri Park, Tae-Jung Park, Namhoo Kim, Jin-Oh Park

**Affiliations:** Department of Orthopedic Surgery, Yongin Severance Hospital, Yonsei University College of Medicine, 363, Dongbaekjukjeon-daero, Giheung-gu, Yongin-si 16995, Gyeonggi-do, Republic of Korea; ysos111@yuhs.ac (H.-S.K.); ncd1896@yuhs.ac (S.-R.P.); xowjd123@yuhs.ac (T.-J.P.); namhoo.kim@yuhs.ac (N.K.)

**Keywords:** biportal endoscopic spine surgery, hidden blood loss, total blood loss, minimally invasive, lumbar interbody fusion

## Abstract

**Background:** Perioperative blood loss in lumbar spine surgery is closely linked to postoperative recovery and complications, yet hidden blood loss (HBL) is often underestimated. This study aimed to compare total and hidden blood loss between biportal endoscopic spine surgery (BESS) and open surgery and to identify perioperative risk factors influencing HBL. **Methods:** This retrospective study included 261 patients who underwent lumbar decompression or fusion between January 2020 and February 2024. Patients were divided by surgical approach, procedure type and number of levels. Total blood loss (TBL), visible blood loss (VBL), and HBL were calculated from hematocrit changes using established formulas. Linear mixed and multivariate regression models were used to assess intergroup differences and identify independent predictors. **Results:** BESS demonstrated significantly reduced intraoperative bleeding and drainage volumes, shorter hospital stay, and earlier ambulation than traditional open surgery. Postoperative hematocrit decline was smaller in BESS group in multi-level decompression and fusion indicating a smaller postoperative decline in the BESS group. Although BESS reduced visible bleeding, HBL remained a major portion of TBL in both groups. Multivariate analysis identified longer operative time, diabetes mellitus, multi-level surgery, and fusion procedures as independent predictors of increased TBL and HBL, while smoking and dorsal extensor thickness were associated with increased loss only in traditional open surgery. **Conclusions:** BESS effectively reduces visible perioperative bleeding and enhances early recovery, but HBL remains clinically significant. Surgical extent and metabolic factors, rather than approach alone, determine overall bleeding, emphasizing the importance of surgical proficiency and meticulous hemostasis.

## 1. Introduction

Perioperative blood loss is a significant concern in lumbar spine surgery as increased blood loss is associated with increased postoperative complications, increased morbidity and mortality, and adverse clinical outcomes including prolonged hospital stays [1]. Therefore, accurately determining the amount of blood loss before and after spinal surgery is crucial. Traditionally, total blood loss (TBL) during spinal surgery is estimated based solely on intraoperative blood loss and postoperative drainage. However, the actual TBL is often considerably higher than that indicated by these measurements. Sehat et al. first introduced the concept of hidden blood loss (HBL), the unmeasured blood loss that occurs due to bleeding into the surrounding tissues and hemolysis, which is often unaccounted for in standard assessments [2]. Similarly, in a study of posterior spine fusion, HBL resulted in a higher TBL than that estimated from pre- and postoperative measurements [3].

In minimally invasive spine surgery, the visible blood loss is minimal, making the accurate determination of TBL even more challenging [4]. Understanding the role of HBL in biportal endoscopic spine surgery (BESS) versus that in open surgery is clinically crucial. Biportal endoscopic spine surgery minimizes muscle and soft tissue damage while reducing blood loss; additionally, it has superior clinical outcomes [5,6]. However, research on blood management processes, such as TBL and HBL, remains insufficient.

Therefore, we hypothesized that BESS would result in lower TBL and HBL than traditional open surgery. In this study, we compared the patterns of perioperative blood loss between BESS and open procedures in patients who underwent lumbar fusion or decompression. We further analyzed the postoperative changes in TBL and HBL over time and sought to identify the clinical and procedural risk factors that may influence the extent of HBL.

## 2. Materials and Methods

### 2.1. Patient Selection

This retrospective study included patients who underwent open or endoscopic surgery for degenerative lumbar disease at the Yongin Severance Orthopedic Department between January 2020 and February 2024. Three spine surgeons performed the surgeries at the same center. One surgeon performed BESS, whereas two surgeons performed traditional open surgery. This study was approved by the Institutional Review Board of Yongin Severance Hospital (approval number: 9-2024-0138). The patients were categorized into eight groups based on the following criteria: type of surgery (decompression surgery or posterior interbody fusion), number of levels involved (single-level or multi-level surgery), and surgical approach (traditional open surgery or BESS). All traditional open surgeries were performed using the unilateral laminectomy bilateral decompression technique. Inclusion criteria were as follows: For decompression procedures, patients were included if they had moderate to severe lumbar spinal stenosis confirmed by magnetic resonance imaging (MRI) and had undergone conservative treatments, including medication, physical therapy, or selective nerve block, for more than 6 weeks without improvement in neurological symptoms. For interbody fusion procedures, patients were included if they were diagnosed with lumbar degenerative disease accompanied by foraminal stenosis, such as spinal stenosis, spondylolisthesis, or degenerative disc disease, and had persistent back or leg pain refractory to conservative treatment for more than 3 months. The exclusion criteria were as follows: (1) previous history of lumbar surgery; (2) surgery performed for infection, tumor, or trauma; (3) use of anticoagulants prior to surgery; and, (4) requirement of transfusion after surgery. Finally, total 261 patients were enrolled in our study.

### 2.2. Surgical Procedure

#### 2.2.1. BESS Technique

BESS was performed with the patient in the prone position under general or epidural anesthesia. A skin incision was made approximately 1 cm medial to the pedicle border, and two portals were created, either higher or lower, around the interlaminar space. An ipsilateral laminotomy was conducted, followed by detachment of the ligamentum flavum to create sufficient space for accessing the traversing root pathway, the lateral area of the dural sac, and the exiting root pathway. The same procedure was subsequently performed on the contralateral side [7].

For BESS interbody fusion, the procedure utilized the same patient positioning and skin incision location over the pedicle area [8]. After central decompression was achieved, ipsilateral total facetectomy was performed, and both the exiting and traversing roots were inspected. Following discectomy and disc preparation, an autologous bone graft was placed in a suitably sized cage, and percutaneous pedicle screws were inserted.

#### 2.2.2. Traditional Open Surgery

Open decompression surgery involved unilateral laminotomy and bilateral decompression. The ipsilateral fascia and paravertebral muscles were separated from the bony structures through a midline incision. Subsequently, ipsilateral laminotomy and ligamentum flavum removal were performed, followed by bilateral decompression and decompression of the traversing roots. Open interbody fusion surgery was performed with the same patient positioning and surgical approach. After bilateral facetectomy with laminectomy, discectomy with disc preparation and cage insertion were performed, followed by the insertion of pedicle screws on both sides.

#### 2.2.3. Perioperative Blood Management

Perioperative blood management was standardized across all patients. Tranexamic acid was routinely administered intravenously before surgery unless contraindicated. Intraoperative systolic blood pressure was maintained at approximately 100–110 mmHg under anesthetic management. In patients undergoing BESS, saline irrigation was delivered via a water tower system using natural gravity, maintaining an average pressure of approximately 30–40 mmHg. All surgical procedures were performed according to standardized operative techniques across surgeons.

### 2.3. Outcome Evaluation

**Figure 1:**
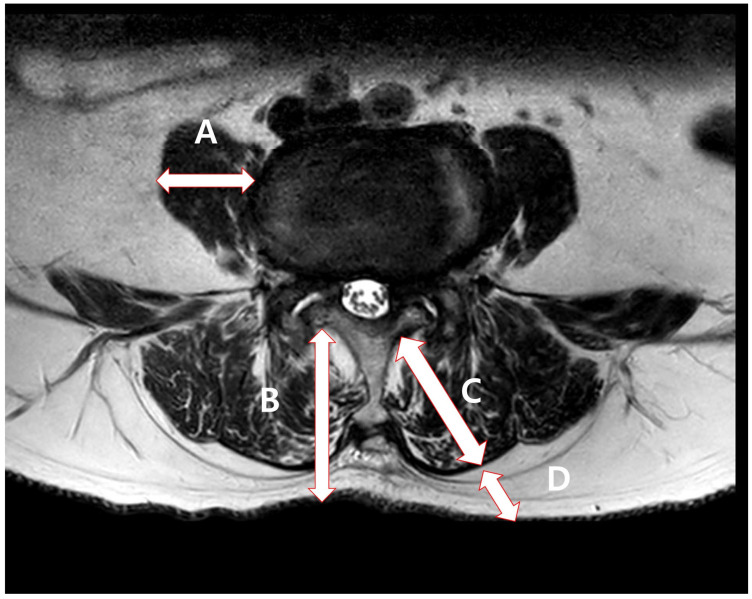
Measurement of soft tissue thickness. Soft tissue thickness was measured using preoperative MRI axial cut at the surgical level. A: Psoas muscle thickness, B: interlaminar thickness, C: Dorsal extensor muscle thickness, D: subcutaneous fat thickness.

Preoperative and daily hematocrit, albumin, prothrombin time (PT) and activated prothromboplastin time (aPTT) levels were measured immediately and on postoperative days (POD) 0 to 3. Clinical outcomes assessed after surgery included back and leg visual analog scale (VAS) scores measured preoperatively and on POD 3, the duration of Jackson–Pratt (JP) or hemovac drainage maintenance, and length of hospital stay. In addition, soft tissue thickness (interlaminar thickness, psoas muscle thickness, dorsal extensor thickness, subcutaneous fat thickness) was measured using the axial cut of the surgical area in lumbar MRI performed preoperatively (Figure 1). The degree of fat infiltration of the paraspinal muscle was classified using the Goutallier grade [9].

Patient blood volume (PBV) was calculated using the formula proposed by Nadler et al. [10].

PBV = height (m)^3^ × K1 + weight (kg) × K2 + K3

(K1 = 0.3669, K2 = 0.03219, K3 = 0.6041 for men and K1 = 0.3561, K2 = 0.03308, K3 = 0.1833 for women).

Further, TBL, visible blood loss (VBL) and HBL were calculated based on changes in hematocrit levels and PBV according to the formula by Gross et al. [11]:

TBL = PBV × (Hct_pre_ − Hct_post_)/Hct_ave_, Hct_ave_ = (Hct_pre_ + Hct_post_)/2

VBL = intraoperative bleeding + postoperative drainage.

HBL = TBL − VBL

### 2.4. Statistical Analysis

Statistical analyses were conducted using SPSS software (version 26.0; IBM Corp., Armonk, NY, USA). Categorical variables were analyzed using the chi-square test, whereas continuous variables were assessed using independent *t*-tests. A linear mixed model was employed to evaluate the TBL, HBL, and changes in hematocrit values over different groups and time points. Preoperative back and leg VAS scores were adjusted, and postoperative VAS scores were compared using analysis of covariance (ANCOVA). To analyze risk factors affecting total blood loss and hidden blood loss, multivariable linear regression was performed on variable factors measured preoperative in the patients included in the study. Statistical significance was set at *p* < 0.05.

## 3. Results

### 3.1. Demographic Data

Across all groups, patients who underwent endoscopic surgery had a significantly longer operation time, but, shorter duration of postoperative drain amount, maintenance and hospital stay. Additionally, the patients in the endoscopic surgery group achieved earlier ambulation than those in the traditional open surgery group (Table 1). On comparing preoperative and postoperative leg and back VAS scores, the postoperative VAS scores were significantly lower in the BESS group across all groups (Table 2).

### 3.2. Change in Hematocrit, TBL and HBL

Changes in hematocrit levels were compared between the BESS and open surgery groups. In both the multi-level decompression and fusion cohorts, hematocrit levels showed a significant group–time interaction (*p* = 0.017 and *p* = 0.026, respectively), indicating a smaller postoperative decline in the BESS group (Table 3). In the decompression cohort, temporal changes in TBL differed between the two techniques. In multi-level decompression, a significant group–time interaction was observed (*p* = 0.045), indicating different trajectories of total blood loss between the BESS and open surgery groups. Although no consistent differences were observed at earlier postoperative time points, TBL values were lower in the BESS group by POD 3 in both single-level (539.7 ± 274.1 mL vs. 650.3 ± 231.7 mL) and multi-level decompression (687.8 ± 314.9 mL vs. 773.8 ± 275.7 mL). For interbody fusion surgery, no significant difference in TBL was observed between the two techniques up to POD 3 (Table 3). HBL showed a consistent tendency toward lower values in the BESS group across all categories. In single-level fusion, patients treated with BESS exhibited significantly lower HBL than those treated with traditional open surgery (182.4 ± 156.2 mL vs. 276.4 ± 180.8 mL, *p* = 0.026), while differences in other subgroups were not statistically significant (Table 3).

### 3.3. Risk Factors Related with TBL and HBL

Baseline and perioperative variables were comparable between the two groups, except that BESS showed a longer operative time but significantly lower intraoperative bleeding and drainage volumes (Table 4).

Univariate and multivariate regression analyses identified several significant predictors of TBL and HBL (Table 5 and Table 6). Longer operative time, diabetes, multi-level surgery, and fusion procedure were independently associated with increased TBL and HBL in both groups (all *p* < 0.05). Among these, operative time showed the strongest linear correlation, while diabetes mellitus remained an independent predictor of increased HBL, with diabetic patients showing an average increase of approximately 100 mL in HBL even after adjustment for confounding variables. In the open surgery group, smoking and greater dorsal extensor thickness were additional independent risk factors, whereas these associations were not observed in the BESS group.

## 4. Discussion

HBL refers to postoperative blood loss that is not directly measurable through intraoperative suction or postoperative drainage. It is thought to occur as a result of hemolysis, fluid shifts, and interstitial pooling of blood within the soft tissues [12,13]. With the advancement of minimally invasive and endoscopic spine surgery, visible intraoperative bleeding has markedly decreased. However, HBL continues to represent a clinically significant proportion of TBL. Particularly in endoscopic procedures with continuous saline irrigation and limited surgical exposure, fluid dynamics and hemostatic mechanisms differ from those in open surgery, warranting more careful assessment of HBL [14,15].

Previous studies consistently reported that endoscopic and minimally invasive techniques significantly TBL and HBL compared with traditional open surgery [16,17]. In a comparative study of single-level interbody fusion, endoscopic TLIF showed lower TBL (approximately 327 mL vs. 428 mL) and HBL (99 mL vs. 271 mL) than traditional open TLIF, yet HBL accounted for a greater proportion of TBL about 70% versus 37% in the traditional open group [18]. Other studies have also reported mean HBL volumes of 360–780 mL, comprising 60–80% of TBL in minimally invasive or fully endoscopic fusion procedures [19,20,21]. In a recent multicenter analysis, BESS demonstrated lower mean TBL (247 mL vs. 299 mL) and HBL (149 mL vs. 171 mL) compared with open decompression, although the differences did not reach statistical significance [22]. These findings suggest that while endoscopic surgery may reduce visible bleeding, HBL remains a substantial component of TBL.

In our study, both intraoperative bleeding and postoperative drainage volumes were smaller in the BESS group than in the traditional open surgery group. The reduction in VBL suggests a potential hemostatic benefit of the BESS technique. This may be attributed to minimized muscle detachment and soft tissue trauma, as well as continuous saline irrigation, which provides a clear field and facilitates micro-hemostasis. Moreover, shorter hospital stays and earlier ambulation observed in the BESS group may reflect improved postoperative recovery associated with reduced visible blood loss. However, while the change in hematocrit from POD 0 to POD 3 was significantly smaller in multi-level BESS patients, absolute TBL and HBL were lower but not statistically different from those in traditional open surgery. These findings indicate that the benefit of BESS cannot be fully evaluated based solely on VBL, and further identification of factors contributing to HBL is warranted.

Regarding risk factors for HBL, previous literature has shown relatively consistent results [14,23,24]. Longer operative time, greater number of fusion levels, and comorbid hypertension have all been associated with increased HBL in spine surgery [19,20,21]. It has been suggested that as operative duration and the number of levels increase, bone and soft tissue damage become more extensive, leading to greater blood loss. In BESS, prolonged irrigation may result in fluid permeation into soft tissue and bone surfaces, contributing to postoperative hemoglobin dilution and hidden loss [12]. To address the limitations of suction-based measurements under continuous irrigation, we adopted a hematocrit-based estimation method for total and hidden blood loss. This approach allows a more physiologic assessment of perioperative blood loss, although it may still be influenced by perioperative fluid shifts. These phenomena may be further influenced by increased exposure of cancellous bone and venous plexuses, as well as delayed vasoconstrictive and hemostatic responses in hypertensive patients. In addition, advanced age, reduced lumbar extensor muscle thickness, increased estimated blood volume, and lower postoperative hemoglobin or fibrinogen levels have also been reported as patient-related risk factors for increased HBL [4,12,19,20,21]. Diabetes has similarly been identified as a risk factor for both TBL and HBL in open and endoscopic spine surgery [4,25]. As a result, these findings suggest that HBL is determined by a complex interplay between surgical factors and patient-specific hematologic and systemic conditions.

Although the observed increase of approximately 100 mL in HBL among diabetic patients may appear modest, it may have clinical relevance in vulnerable populations, particularly in elderly patients with limited physiologic reserve. Even small additional hidden blood loss could contribute to postoperative hemodynamic instability, such as orthostatic hypotension, warranting closer postoperative monitoring in this subgroup.

In our multivariable analysis, diabetes, longer operative time, fusion procedure, and multi-level surgery were significant risk factors for both TBL and HBL in both surgical techniques. Meanwhile, smoking, interlaminar thickness, and dorsal extensor muscle thickness were independent risk factors only in the traditional open surgery group. The degree of fatty degeneration, evaluated by the Goutallier grade, did not significantly influence blood loss. These findings suggest that although BESS may confer advantages by minimizing paraspinal muscle injury compared with traditional open surgery, meticulous surgical technique remains essential. Controlling operative duration, fusion extent, and the number of surgical levels is critical even in BESS to achieve optimal hemostasis. Preoperative PT and aPTT were not associated with perioperative blood loss, likely because all included patients had normal preoperative coagulation profiles and those taking anticoagulants were excluded.

This study has several limitations. First, its retrospective design introduces potential selection bias, as all procedures were performed at a single institution. Second, accurate quantification of intraoperative bleeding during continuous saline irrigation remains technically challenging. Third, variability in the timing of postoperative blood sampling may have affected the calculation of HBL. Finally, the difference in the proportion of multilevel cases between groups may have partially influenced the results. In addition, despite the use of standardized surgical techniques, potential surgeon-specific variability may have influenced intraoperative blood loss. Perioperative management was applied according to routine clinical practice, and the absence of a fully standardized blood management protocol may have affected the assessment of hidden blood loss. Future multicenter prospective studies with standardized blood sampling intervals and quantitative irrigation analysis are needed to establish more precise estimation methods and identify modifiable factors contributing to hidden blood loss in spine surgery.

## 5. Conclusions

In this study, BESS demonstrated significantly lower intraoperative bleeding and postoperative drainage compared with traditional open surgery, reflecting reduced visible perioperative bleeding. However, hidden blood loss remained a substantial component of total blood loss regardless of surgical approach. Multivariate analysis showed that operative time, diabetes mellitus, surgical extent, and fusion procedures were the primary determinants of increased blood loss, rather than the approach itself. These findings underscore the need for careful postoperative hemodynamic monitoring, high level of surgical expertise, careful management of metabolic conditions and meticulous surgical technique, even when performing BESS.

## Figures and Tables

**Table 1:** Demographic data.

Decompression Surgery						
	Single-Level			Multi-Level		
	BESS	Open Surgery	*p*-Value	BESS	Open Surgery	*p*-Value
Patients	42	42		32	33	
Age (years)	63.38 ± 8.63	64.52 ± 12.02	0.618	67.31 ± 10.50	68.06 ± 7.59	0.742
DM	12	12	1	9	9	0.938
HTN	18	19	0.826	17	19	0.718
BMI	25.95 ± 3.80	26.41 ± 3.51	0.573	26.63 ± 3.99	26.30 ± 4.28	0.751
Operation time (min)	74.88 ± 11.03	64.78 ± 18.13	0.003 *	99.68 ± 15.32	79.78 ± 19.10	<0.001 *
Intraoperative bleeding (mL)	54.76 ± 20.39	55.71 ± 43.95	0.899	79.39 ± 69.56	81.31 ± 49.95	0.899
Drainage amount (mL)	47.54 ± 28.53	72.69 ± 44.95	0.003 *	103.96 ± 82.15	156.84 ± 73.20	0.007 *
Mean drain maintenance (day)	1	1.40 ± 0.544	<0.0001 *	1.16 ± 0.45	1.73 ± 0.57	<0.0001 *
Preoperative Hematocrit (%)	41.62 ± 3.52	40.14 ± 1.49	0.081	40.13 ± 4.60	41.04 ± 3.47	0.368
Postoperative hospital day (day)	3.85 ± 1.08	4.66 ± 1.32	0.003 *	4.37 ± 1.04	5.82 ± 2.29	0.001 *
**Interbody Fusion Surgery**						
	**Single-Level**			**Multi-Level**		
	**BESS**	**Open Surgery**	***p*** **-Value**	**BESS**	**Open Surgery**	***p*** **-Value**
Patients	34	29		24	25	
Age (years)	65.44 ± 6.23	67.10 ± 7.93	0.355	70.20 ± 7.13	70.92 ± 11.12	0.79
DM	12	12	1	8	9	0.845
HTN	17	19	0.62	11	14	0.477
BMI (T-score)	25.10 ± 3.10	25.17 ± 3.08	0.922	25.18 ± 2.79	25.08 ± 3.05	0.909
Operation time (min)	157.55 ± 23.73	125.93 ± 19.01	<0.0001 *	222.12 ± 23.83	162.68 ± 36.25	<0.0001 *
Intraoperative bleeding (mL)	123.97 ± 44.04	189.93 ± 88.03	0.007 *	193.91 ± 18.50	251.60 ± 70.86	0.005 *
Drainage amount (mL)	122.64 ± 42.34	169.41 ± 48.43	0.0001 *	152.37 ± 17.12	235.32 ± 63.87	<0.0001 *
Mean drain maintenance (day)	1	2.21 ± 0.62	<0.0001 *	1.17 ± 0.38	2.32 ± 0.48	<0.0001 *
Preoperative Hematocrit (%)	37.90 ± 2.90	39.25 ± 3.89	0.119	40.25 ± 3.76	40.47 ± 3.28	0.83
Postoperative hospital day (day)	7.03 ± 3.91	8.38 ± 2.90	0.03 *	8.62 ± 1.25	9.84 ± 2.94	0.03 *

DM: Diabetes mellitus, HTN: Hypertension; Statistically significant values (*p* < 0.05) are highlighted in bold and marked with asterisk.

**Table 2:** Comparison of clinical outcomes after surgery in BESS and open surgery.

	BESS (SE)	Traditional Open Surgery (SE)	*p*-Value
**Decompression Single level**			
Post op Back VAS score	4.45 ± 0.77	5.19 ± 0.71	<0.0001 *
Post op Leg VAS score	2.77 ± 0.54	2.63 ± 1.00	0.88
**Decompression Multi-level**			
Post op Back VAS score	4.81 ± 0.82	5.43 ± 0.83	0.003 *
Post op Leg VAS score	2.83 ± 0.56	2.57 ± 1.04	0.76
**Interbody Fusion Single level**			
Post op Back VAS score	4.20 ± 0.66	4.89 ± 0.86	0.003 *
Post op Leg VAS score	2.98 ± 1.11	2.83 ± 0.61	0.66
**Interbody Fusion Multi-level**			
Post op Back VAS score	5.23 ± 0.72	6.38 ± 0.76	0.001 *
Post op Leg VAS score	2.88 ± 1.21	2.81 ± 0.56	0.87

Statistically significant values (*p* < 0.05) are highlighted in bold and marked with asterisk.

**Table 3:** Changes in hematocrit, TBL and HBL between preoperative and postoperative day.

Change in Hematocrit						
	Single-Level Decompression, Estimated Mean (SE)			Multi-Level Decompression, Estimated Mean (SE)		
	BESS	Open Surgery	Group × Time *p*-Value	BESS	Open Surgery	Group × Time *p*-Value
Preoperative	41.624 (0.594)	40.140 (0.594)	0.188	40.131 (0.719)	41.045 (0.708)	0.017 *
POD 1	37.812 (0.583)	36.233 (0.583)		35.550 (0.678)	36.327 (0.667)	
POD 3	36.848 (0.621)	34.531 (0.621)		34.178 (0.664)	33.909 (0.654)	
	**Single-Level Interbody Fusion, Estimated Mean (SE)**			**Multi-Level Interbody Fusion, Estimated Mean (SE)**		
	**BESS**	**Open Surgery**	**Group × Time** ***p*** **-Value**	**BESS**	**Open Surgery**	**Group × Time** ***p*** **-Value**
Preoperative	37.900 (0.582)	39.255 (0.630)	0.122	40.254 (0.720)	40.472 (0.706)	0.026 *
POD 1	32.641 (0.551)	32.448 (0.597)		33.217 (0.680)	32.440 (0.667)	
POD 3	30.662 (0.563)	30.669 (0.610)		30.992 (0.719)	29.740 (0.705)	
**Change in Total Blood Loss**						
	**Single-Level Decompression, Estimated Mean (SE)**			**Multi-Level Decompression, Estimated Mean (SE)**		
	**BESS**	**Open Surgery**	**Group × Time** ***p*** **-Value**	**BESS**	**Open Surgery**	**Group × Time** ***p*** **-Value**
POD 0	192.030 (38.844)	218.528 (38.844)	0.121	239.933 (51.977)	300.441 (51.183)	0.045 *
POD 1	419.222 (32.730)	438.198 (32.730)		515.135 (47.498)	494.373 (46.773)	
POD 3	539.711 (39.161)	650.256 (39.161)		687.749 (52.263)	773.801 (51.465)	
	**Single-Level Interbody Fusion, Estimated Mean (SE)**			**Multi-Level Interbody Fusion, Estimated Mean (SE)**		
	**BESS**	**Open Surgery**	**Group × Time** ***p*** **-Value**	**BESS**	**Open Surgery**	**Group × Time** ***p*** **-Value**
POD 0	371.199 (44.721)	469.191 (48.423)	0.685	472.342 (43.688)	568.705 (42.805)	0.476
POD 1	589.590 (52.328)	751.836 (56.659)		741.631 (43.688)	874.244 (42.805)	
POD 3	835.741 (61.449)	980.913 (66.536)		1010.037 (43.688)	1202.940 (42.805)	
**Change in Hidden Blood Loss**						
	**Single-Level Decompression, Estimated Mean (SE)**			**Multi-Level Decompression, Estimated Mean (SE)**		
	**BESS**	**Open Surgery**	**Group × Time** ***p*** **-Value**	**BESS**	**Open Surgery**	**Group × Time** ***p*** **-Value**
POD 0	137.268 (39.392)	162.814 (39.392)	0.447	158.620 (54.738)	221.048 (53.903)	0.650
POD 1	316.912 (31.885)	382.484 (31.885)		329.854 (42.836)	336.554 (42.182)	
POD 3	437.401 (33.849)	521.851 (33.849)		502.467 (43.157)	537.559 (42.498)	
	**Single-Level Interbody Fusion, Estimated Mean (SE)**			**Multi-Level Interbody Fusion, Estimated Mean (SE)**		
	**BESS**	**Open Surgery**	**Group × Time** ***p*** **-Value**	**BESS**	**Open Surgery**	**Group × Time** ***p*** **-Value**
POD 0	247.229 (44.141)	279.260 (47.795)	0.026 *	278.425 (53.371)	317.105 (52.293)	0.419
POD 1	342.973 (46.824)	477.198 (50.700)		395.339 (38.076)	504.984 (37.307)	
POD 3	589.124 (53.492)	621.568 (57.920)		663.746 (30.715)	716.020 (30.094)	

Statistically significant values (*p* < 0.05) are highlighted in bold and marked with asterisk.

**Table 4:** Risk factor parameters between BESS and traditional open surgery.

Variables	BESS	Traditional Open Surgery	*p*-Value
Patient Number (n)	132	129	
Fusion surgery (n)	58	54	
Multi-level (n)	56	58	
Gender (F/M)	68/64	67/52	0.94
BMI	25.76	25.85	0.83
Diabetes mellitus	41	42	0.79
Hypertension	63	68	0.42
Smoking	33	33	0.59
Postop mean Blood pressure (mmHg)	127.33	128.67	0.26
Operation time (min)	128.96	101.34	<0.001 *
Intraoperative bleeding (mL)	104.32	129.9	0.016
Postoperative drain amount (cc)	99.62	147.48	<0.001 *
Preoperative Hct (%)	40.05	40.23	0.7
Preoperative serum albumin (g/dL)	4.44	4.47	0.38
Preoperative serum PT (s)	0.94	0.93	0.94
Preoperative serum aPTT (s)	27.2	26.7	0.16
Interlaminar thickness (mm)	43.36	42.86	0.56
Psoas muscle thickness (mm)	33.32	31.87	0.11
Dorsal extensor muscle thickness (mm)	37.26	37.94	0.43
Subcutaneous fat thickness (mm)	12.42	12.88	0.51
Goutallier grade	1.77	2.06	0.052

Statistically significant values (*p* < 0.05) are highlighted in bold and marked with asterisk.

**Table 5:** Results of univariate regression for TBL and HBL.

Variable	BESS				Traditional Open Surgery			
	Total blood Loss		Hidden Blood Loss		Total Blood Loss		Hidden Blood Loss	
	β (SE)	*p*-Value	β (SE)	*p*-Value	β (SE)	*p*-Value	β (SE)	*p*-Value
Age	1.36 (3.53)	0.70	−1.40 (3.02)	0.64	3.10 (2.80)	0.07	2.03 (1.93)	0.07
Diabetes mellitus	249.86 (61.79)	<0.001 *	197.96 (48.74)	<0.001 *	284.87 (56.68)	<0.0001 *	221.13 (37.71)	<0.0001 *
Hypertension	−6.92 (60.74)	0.91	−12.87 (47.92)	0.79	6.70 (58.24)	0.91	−15.30 (39.87)	0.70
Goutallier grade	−16.16 (26.08)	0.54	−24.62 (20.69)	0.24	−10.01 (25.81)	0.70	−14.01 (17.65)	0.43
BMI	−8.70 (8.59)	0.31	−5.95 (6.78)	0.38	4.48 (8.16)	0.58	7.24 (5.56)	0.20
Smoking	101.84 (84.08)	0.96	114.26 (65.97)	0.09	247.14 (70.88)	<0.0001 *	208.11 (47.34)	<0.0001 *
Postoperative mean BP (mmHg)	−0.13 (2.990)	0.35	−0.71 (2.35)	0.76	−2.11 (3.19)	0.51	0.93 (2.19)	0.67
Operation time (min)	3.99 (0.39)	**<0.0001**	2.42 (0.36)	<0.0001 *	5.95 (0.39)	<0.0001 *	3.05 (0.35)	<0.0001 *
Intraoperative bleeding (mL)	2.45 (0.44)	<0.0001 *	1.07 (0.38)	<0.0001 *	1.54 (0.24)	<0.0001 *	0.25 (0.19)	0.008 *
Interlaminar thickness (mm)	5.55 (4.18)	0.18	4.56 (3.62)	0.20	2.42 (1.55)	0.03 *	2.16 (1.14)	0.03 *
Dorsal extensor thickness (mm)	−3.79 (4.54)	0.40	−1.41 (2.58)	0.55	10.59 (4.13)	<0.0001 *	7.11 (2.95)	0.017 *
Subcutaneous fat thickness (mm)	−3.16 (5.45)	0.56	−1.61 (3.58)	0.65	−2.61 (5.96)	0.66	−4.56 (4.04)	0.26
Psoas muscle thickness (mm)	7.96 (4.11)	0.06	7.37 (3.18)	0.09	1.88 (4.00)	0.64	2.39 (2.74)	0.07
Preoperative Albumin (g/dL)	19.99 (88.30)	0.82	81.59 (69.33)	0.24	55.52 (91.44)	0.54	94.63 (62.16)	0.13
Preoperative PT (s)	−389.64 (517.67)	0.45	−171.63 (409.14)	0.68	−1237.88 (536.58)	0.23	−560.03 (371.86)	0.13
Preoperative aPTT (s)	−3.96 (12.28)	0.75	−1.77 (9.69)	0.86	−22.64 (10.94)	0.14	−10.09 (7.57)	0.18
Level								
Single-level	ref		ref		ref		ref	
Multi-level	153.72 (59.89)	0.011 *	66.30 (48.09)	0.017 *	173.46 (56.39)	<0.0001 *	51.90 (39.77)	0.019 *
Procedure								
Decompression surgery	ref		ref		ref		ref	
Interbody fusion surgery	304.13 (55.01)	<0.0001 *	154.75 (50.01)	<0.0001 *	379.08 (48.81)	<0.0001 *	136.53 (38.51)	<0.0001 *

Statistically significant values (*p* < 0.05) are highlighted in bold and marked with asterisk.

**Table 6:** Results of multivariate linear regression for TBL and HBL.

Variable	BESS				Traditional Open Surgery			
	Total Blood Loss		Hidden Blood Loss		Total Blood Loss		Hidden Blood Loss	
	β (SE)	*p*-Value	β (SE)	*p*-Value	β (SE)	*p*-Value	β (SE)	*p*-Value
Diabetes mellitus	118.68 (45.35)	0.01 *	95.49 (39.28)	0.016 *	135.23 (36.87)	<0.0001 *	121.43 (32.45)	<0.0001 *
Smoking	61.34 (63.07)	0.33	61.70 (35.46)	0.33	141.57 (47.37)	<0.0001 *	107.20 (39.73)	<0.0001 *
Operation time (min)	8.56 (1.06)	**<0.00001**	8.46 (1.063)	<0.00001 *	6.11 (0.68)	<0.0001 *	4.72 (0.57)	<0.0001 *
Intraoperative bleeding (mL)	0.83 (0.56)	0.15	0.82 (0.57)	0.14	00.63 (0.24)	0.27	−0.31 (0.20)	0.12
Interlaminar thickness (mm)	5.55 (4.18)	0.18	4.56 (3.62)	0.20	2.42 (1.55)	0.03 *	2.16 (1.14)	0.03 *
Dorsal extensor thickness (mm)	0.33 (2.74)	0.82	0.51 (3.24)	0.87	2.35 (1.29)	0.04 *	1.97 (0.76)	0.007 *
Level								
Single-level	ref		ref		ref		ref	
Multi-level	238.95 (64.53)	<0.0001 *	249.56 (55.90)	<0.0001 *	259.64 (38.96)	0.008 *	343.21 (62.67)	0.008 *
Procedure								
Decompression surgery	ref		ref		ref		ref	
Interbody fusion surgery	555.27 (117.39)	<0.0001 *	54.33 (101.70)	<0.0001 *	156.39 (71.81)	<0.0001 *	165.64 (60.22)	0.006 *

Statistically significant values (*p* < 0.05) are highlighted in bold and marked with asterisk.

## Data Availability

The datasets generated and/or analyzed during the current study are not publicly available due to ethical and privacy concerns but are available from the corresponding author on reasonable request.

## Abbreviations

The following abbreviations are used in this manuscript:

BESS	Biportal endoscopic spine surgery
TBL	Total blood loss
HBL	Hidden blood loss
VBL	Visible blood loss
PBV	Patient blood volume
Hct	Hematocrit
PT	Prothrombin time
aPTT	Activated Partial Thromboplastin Time
POD	Postoperative days
VAS	Visual analog scale
